# Binocular Eye Alignment Monitor (BEAM): Home Monitoring Proof-of-Concept Device to Quantify Binocular Alignment for Intermittent Exotropia

**DOI:** 10.1167/tvst.14.8.17

**Published:** 2025-08-14

**Authors:** Christopher J. Morris, Erin Jenewein, Mitchell Scheiman, Tara L. Alvarez

**Affiliations:** 1 Department of Biomedical Engineering, New Jersey Institute of Technology, Newark, NJ, USA; 2 Pennsylvania College of Optometry, Salus at Drexel University, Elkins Park, PA, USA

**Keywords:** eye movement, intermittent exotropia, home monitoring

## Abstract

**Purpose:**

Intermittent exotropia (IXT) control is challenging to assess clinically because of the variability inherent to this condition. The Binocular Eye Alignment Monitor (BEAM) is a proof-of-concept device that extends eye alignment monitoring for use at home. This study seeks to determine whether the BEAM is feasible for continuously monitoring eye position to identify differences between participants with normal binocular vision and those with IXT.

**Methods:**

An eye tracking system integrated with a custom controller was engineered to measure the magnitude and duration of exodeviation for up to four hours. Twelve participants (six diagnosed with IXT and six with normal binocular vision) completed two separate recordings alongside a clinical sensory motor examination.

**Results:**

The BEAM successfully recorded eye alignment for 90 minutes and demonstrated that IXT participants exhibited significantly greater ocular misalignment than participants with normal binocular vision.

**Conclusions:**

The BEAM is feasible for assessing the magnitude and frequency of temporal exodeviation in the IXT population over prolonged periods while watching a movie from a distance of 6 m, with the participant seated.

**Translational Relevance:**

At-home monitoring to evaluate ocular exodeviation for intermittent exotropic patients is feasible with the BEAM. The BEAM's measurements have the potential to provide objective diagnostic criteria and baseline and outcome assessments for studying the effectiveness of therapeutic interventions in the IXT population.

## Introduction

Intermittent exotropia (IXT) typically appears between 24 and 72 months of age and is an exodeviation characterized by an intermittent outward turn of one eye.^1^^–^^3^ There is no consensus on the disorder's etiology, but theories suggest that muscular imbalance, innervational imbalance, and sensory differences could be the cause.^4^^,^^5^ The predominant symptoms associated with IXT are diplopia (double vision), asthenopia (eye strain), and closure of one eye when exposed to bright sunlight.^5^ Although some patients experience few symptoms, most are concerned with the cosmetic appearance of the eye turn.^5^ The appearance of an eye turn is a significant concern in patients with IXT because of potential psychological impacts, including loss of self-confidence, social isolation, and further psychiatric disorders like depression.^6^^–^^8^ Treatment options for IXT include eye muscle surgery to improve ocular alignment, vision therapy to enhance sensory fusion and neuromuscular control over the eyes, patching one eye, wearing a prism, wearing an overminus lens, or a combination of these treatments.^9^^–^^12^ A major issue in treating IXT is the relatively low success rate of surgical treatment and frequent recurrence of the deviation with long-term follow-up.^13^^,^^14^ Surgical interventions have a long-term success rate of less than 50%, depending on the specific surgery, whereas vision therapy options have a wide range of success rates. Still, none have an effectiveness above 60%.^1^^,^^15^ It is hard to compare the efficacy of surgical and nonsurgical treatment because there is a lack of a unifying metric. Surgical interventions improve the appearance of the IXT by reducing the magnitude of the deviation. Meanwhile, nonsurgical treatment improved the appearance of the IXT by enhancing control of the deviation, reducing the time the eye deviates, but did not significantly alter the size of the deviation.

A major challenge with current diagnostic methodologies is that the frequency of the manifest deviation is the most appropriate measure of the severity of the condition. However, medical devices currently do not assess prolonged recording of eye deviation. Furthermore, a true quantification of the frequency of eye deviation and the duration of the deviation is not available to monitor the deviation throughout the day.^16^ Currently, clinicians use single-time point measurements over a short period in a clinical setting. The frequency of the IXT in the home setting is unknown.^1^^,^^5^^,^^16^ If the patient were in a more relaxed setting, doing a task they are more comfortable with, there may be a difference in the presentation of the exotropia compared to the assessment performed in a clinical setting within a few minutes. Prior studies have shown that fatigue, lack of attentiveness, and exposure to sunlight can cause the exodeviation to become manifest.^1^ However, in the context of currently available clinical diagnostic procedures, exodeviation information is not available to clinicians except for reports from parents or other family members.^17^ IXT intervention success rates are disparate due to the extremely varied nature of the disorder.^15^^,^^18^ Current clinical standards measure the magnitude of the deviation via the prism and alternate cover test, and control of the eye deviation is done with a control score test, predominantly the Office or Newcastle Control Score.^19^^,^^20^ Although these tests provide clinically relevant data, they cannot provide information about the status of the exodeviation throughout the day. Although standard eye tracking technologies exist, commercially available eye trackers lack data analytics to assess IXT for the metrics that clinicians find valuable for monitoring IXT.

To address the lack of a temporal component in the clinical evaluation of IXT, we have developed the Binocular Eye Alignment Monitor (BEAM). It uses a microcomputer, touch screen, and a set of eye-tracking spectacles to unobtrusively monitor the position of each eye independently, within a system that can be easily transported, operated, and used within a home setting. The BEAM offers clinicians continuous monitoring of IXT while the participant is engaged in a typical daily activity, providing diagnostic utility and assessment of therapeutic interventions. This study aims to assess the validity of the BEAM in differentiating participants with normal binocular vision from those with IXT and to determine the frequency of exodeviation over prolonged periods.

## Methods

### Participants

All participants signed written assent, and their legal guardians signed written consent approved by the Institutional Review Boards of the New Jersey Institute of Technology and Pennsylvania College of Optometry, Salus at Drexel University, respectively.

### Hardware Design

The BEAM quantifies the temporal variation of eye position deviation over time. In a home setting, the BEAM monitors horizontal eye position for each eye, quantified monocularly, for up to four hours using a noninvasive, portable device. The user interface is programmed to change the recording time from one minute to four hours. Because the participants were children, all testing was scheduled for 90 minutes, which is the typical duration of a children's movie.

**Figure 1.**
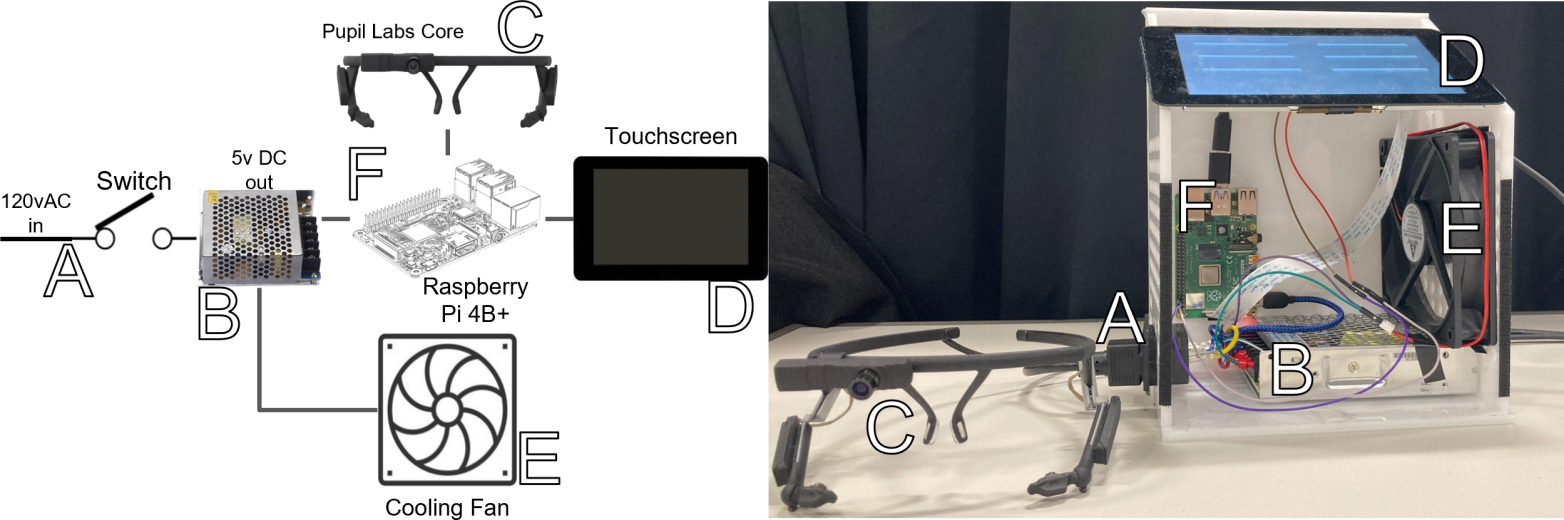
BEAM component layout and physical implementation; (**A**) 120V AC input current and on/off switch; (**B**) 120V AC to 5V DC converter to power components; (**C**) Pupil Labs Core headset with custom camera arms; (**D**) Raspberry Pi touch screen to control system; (**E**) fan to cool system to prevent thermal throttling; and (**F**) Raspberry Pi 4b+ microcomputer to run the system.

The physical architecture is illustrated in Figure 1, with the control box located on the left and the headset worn by an individual on the right. The control box contains power, cooling, and computational components that allow the BEAM to function. The BEAM is controlled by a Raspberry Pi 4B+ microcomputer, which is low in cost (Raspberry Pi, Cambridge, UK). The BEAM runs using a standard 120-volt (V) alternating current (AC) electricity connector, which is converted to 5V direct current (DC) and then separated to power the Raspberry Pi 4B+ and fan (GDSTIME Technology, Shenzhen, China) for cooling the system. A Raspberry Pi 7ʺ touch screen controls the system via a custom user interface. The control box is housed in a quarter-inch acrylic that is laser-cut, and the pieces are cemented together.

**Figure 2.**
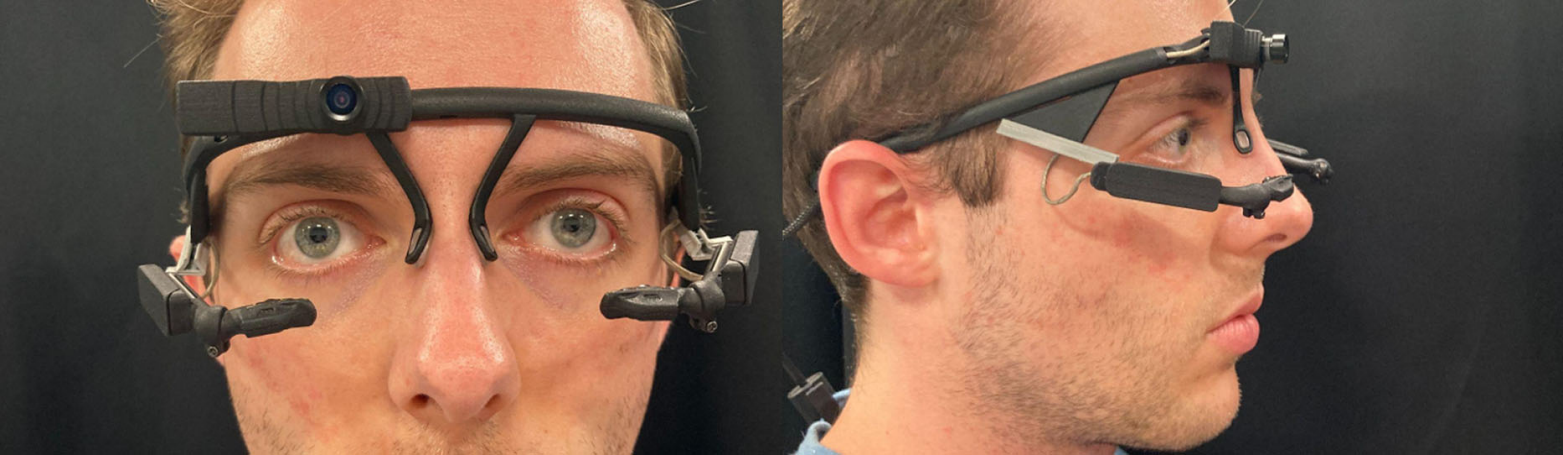
Frontal and sagittal view of an individual wearing the headset component of the BEAM.

The headset, seen in Figure 2, uses the Pupil Labs Core (Berlin, Germany) platform to collect and transmit the video of each eye to the control unit, with custom three-dimensional printed arms to position the cameras for each participant for the eye tracking system to monitor eye position and ensure the participant's vision is not disrupted. The Pupil Labs Core headset weighs 22.75 grams, and the arms weigh less than 5 grams each, allowing a participant to wear the headset for extended periods.

Calibration was performed to convert eye rotation from pixel values to prism diopters (PD, Δ) using a custom calibration board placed 6 meters away from a participant centered along the midsagittal plane. The calibration board contained six light-emitting diodes (LEDs) located at 5, 10, and 15Δ to the left and right visual fields. The calibration sequence was standardized where the participant fixated on the 5Δ, then 10Δ, and then 15Δ LED located in the right visual field for three seconds for each visual target. The sequence was then repeated for the left visual field. The software then averaged the three seconds of eye position sampled at 120 Hz, corresponding to a temporal resolution of 8 msec. The empirically measured spatial resolution of the BEAM is 2Δ. During post-processing, these data were used to create a coefficient for each participant, converting from pixels to Δ.

### Software Design

**Figure 3.**
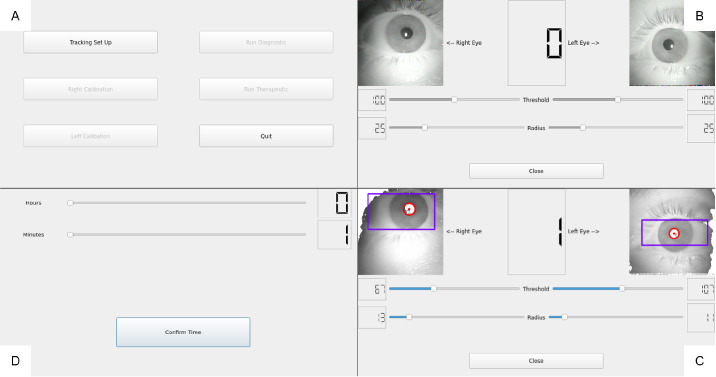
(**A**) Home screen; (**B**) Camera orientation and alignment screen; (**C**) Eye tracking adjustment screen; and (**D**) Set test time screen.

All of the software for the BEAM is custom-written, using C++ version 17, to maintain the video frame rate of 120Hz. The software uses two components: the back end, which controls hardware integration and is responsible for all the processing, and the front end, where the operator can adjust settings to control the BEAM. A touchscreen is integrated to facilitate the BEAM's portability—a user interface (UI) that removes the need for a keyboard and mouse. The QT 5.0 UI development library for C++ version 17 was used to interact with the touch screen. There were four major components in the UI, shown in Figure 3. Figure 3A (top left) is the home screen where the operator controls the sequence of steps in the testing via six buttons. Figure 3B (upper right) is what the operator sees after powering on the BEAM, allowing them to ensure both eyes are positioned properly in the frame. Figure 3C (lower right) enables control of the eye tracking. Each eye image is displayed in the orientation where the operator faces the individual. The software sliders adjust the binary threshold to adjust the contrast of each eye's image and the radius of the pupil's circle. The bounding box is set by tapping on each eye display to specify the box's top left and bottom right coordinates. The values set by each slider are displayed on the outside of each slider. The number in the center displays the minutes that have elapsed since the test began. Figure 3D (bottom left) shows where the operator sets the test time via the minute and hour sliders and where the corresponding numerical values are displayed. The BEAM can collect data from one minute to four hours. The UI and the entire device were developed to ensure ease of use for end users.

**Figure 4.**
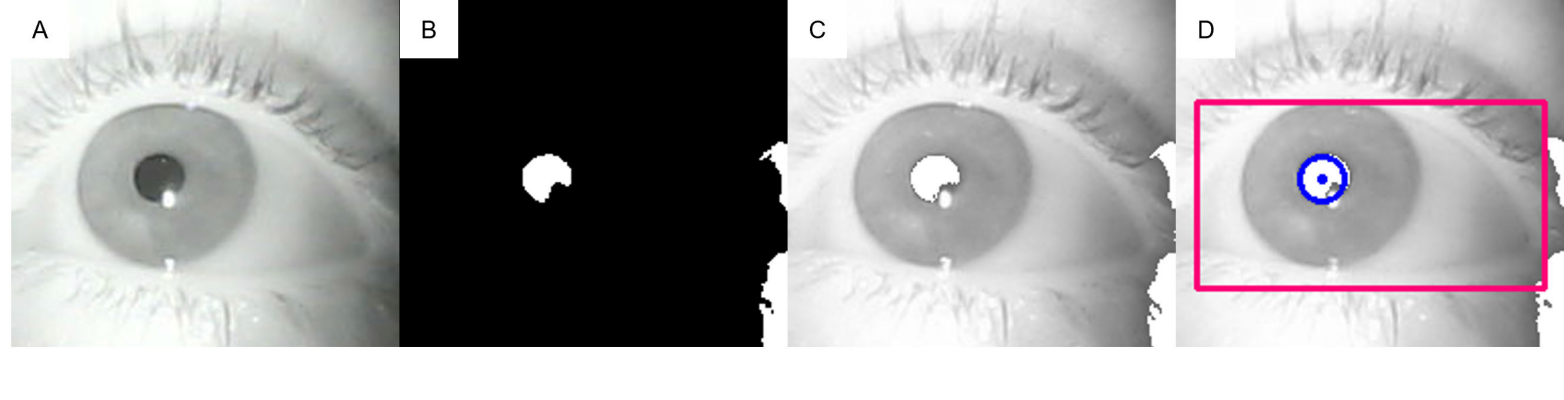
Steps in frame processing: (**A**) Raw image; (**B**) Binary mask to locate Pixel; (**C**) Binary mask applied to gray image; (**D**) Pupil tracking via Hough Circle Transform (*blue circle*) within gaited area (*red rectangle*).

The back end uses OpenCV 4.6.0 and libuvc 0.0.7.^21^^,^^22^ The software first initiates the data streams from the two cameras facing the eyes and ensures the controller receives a signal from each camera. It then waits for the operator to select an input from the UI and initiates recording. Once the operator selects an option, the back end initiates frame acquisition from each camera every 8 milliseconds. It applies the custom image processing algorithm to each frame to calculate the centroid of the pupil. Figure 4 illustrates the pipeline for processing video images. First, the frame is converted to greyscale (Fig. 4A), and then a darkness threshold, set by the operator using the UI, is applied to generate a binary mask (Fig. 4B). The binary mask leverages the absorbency of the pupil compared to the reflectivity of the surrounding iris and sclera, as the pupil appears as a darker area due to its absorption of infrared light (850 nm). In contrast, the rest of the surrounding tissues will reflect the infrared light, which is shown as white (Fig. 4B). The back end then uses the Hough Circle Transform to detect circles of a radius size set by the operator in the UI, under the assumption that the pupil will be approximately circular.^23^ Once this circle is calculated, the center's horizontal (x position) and vertical (y position) are written to a file. This process is repeated for the other eye; then, the back end repeats the algorithm for each subsequent frame within the UI-specified recording time. With the pupil being the center of the eye, the tracking system will record this movement as the eye moves. The algorithm for pupil tracking is used for the six calibration points and the entire eye position recording session.

### Function Testing

The BEAM's operation requires two individuals: a participant (for example, a person with IXT or a person with normal binocular vision) whose eye position is monitored, and an operator who uses the control box to operate the system. The first step is to situate the headset on the participant, ensuring they are comfortable, and the strap is tight enough to secure the headset in place. The BEAM is then powered on. The first screen presents a keyboard to input a file name. After the file name is entered, the operator is presented with a screen showing the raw video feed from the cameras. Within this screen, the operator can adjust the headset's positioning. The operator can also adjust the camera arms to ensure the cameras are properly positioned to acquire frames suitable for eye tracking.

The operator is then presented with the home screen to control the BEAM. Most of the buttons are inactive to ensure that the BEAM is operated in the correct order and becomes accessible when the software is waiting to receive the required input. The first step from the home screen is to set up the eye positional tracking. The operator will adjust the threshold and radius to accurately track the pupil. Once the tracking is properly set, the calibration procedure begins. To ensure proper calibration, the operator instructs the participant to open their eyes and keep them open for a three-second duration, fixating both eyes on the calibration visual cue (the LED visual target) until each step is complete. The head should be kept in a similar position throughout the calibration steps to reduce drift in the calibration. After completing calibration, the diagnostic mode is selected. Here, two more sliders are used to adjust the time of the recording period to hours or hours and minutes. The BEAM then records the data for a set period. Once the test time elapses, the calibration sequence is repeated, and the BEAM testing session ends. The analysis software generates a calibration coefficient for both eyes by averaging the pixel position at each calibration point. The final calibration value is then applied to the raw data, which is filtered using a fourth-order Butterworth filter with a cutoff frequency of 5 Hz.

After the eye movement testing, the filtered data is detrended via a linear regression over the entire eye position recording. A data analyst visually inspects the eye position traces to recenter each eye positional trace, ensuring that each eye position is centered. This is achieved by using the portion of the eye position where the eyes are binocularly aligned, rather than the exodeviated eye position. Once the data has been processed, the left eye position is subtracted from the right eye position to calculate the difference between eye rotation or deviation.

After calibrating, filtering, and detrending the eye movement position, the eye movement data is then processed through the exodeviation detection algorithm. The exodeviation detection algorithm searches for areas where the deviation exceeds a threshold set by a clinician and can be adjusted during post-processing. After deviation threshold detection, the algorithm searches for instances where the eye position is realigned, specifically when the eye position falls below the threshold. The time of deviation is measured between the exodeviation start and end indices. Once the deviation detection algorithm completes the dataset analysis, the length of each deviation in seconds and the maximum deviation within each deviation are calculated. For multiple exodeviations, which are expected in IXT patients, the maximum exodeviation within each loss of fusion is assessed. The BEAM design process for both software and hardware components is informed by input from clinicians who will use the device, ensuring ease of use.

### Sensory Motor Optometric Exam

A residency-trained pediatric optometrist performed the clinical examination for each participant. The sensory motor examination protocol is consistent with the methodology used in other previous and ongoing randomized clinical trials on IXT and other binocular vision disorders.^24^^–^^27^ After collecting demographic information, lensometry is conducted to confirm a participant's spectacles were within tolerance (based on a cycloplegic refraction performed within six months of the visit), and monocular Snellen visual acuity testing is performed to ensure the participant has at least 0.1 logMAR visual acuity in each eye. A unilateral cover test (UCT) determines whether a manifest strabismus (heterotropia) is present. For the UCT, the examiner covered one eye and observed the refixation in the uncovered eye. If the uncovered eye changes fixation when the fixating eye is covered, then heterotropia is present. The magnitude of the heterotropia was then quantified with the prism and alternating cover test (PACT) measurement, which assesses the dissociated magnitude of ocular deviation measured near and distance using a 20/30 letter target (Gulden Fixation Stick at near; isolated Snellen letter at distance). The magnitude of the deviation is measured by neutralizing the ocular deviation with a prism. The UCT and PACT are performed on each participant at a distance (6 m) and near (40 cm), both before and after the BEAM recording.

The control of IXT to assess exodeviation was assessed using the IXT Office Control Scale (OCS) at distance (6 m) and near (40 cm).^19^ The OCS was performed by first observing the participant for 30 seconds at distance or near while the participant was viewing a 20/30 letter target and determining if a manifest strabismus was present; if the examiner noted the eye turn, they scored the amount of time turned on a scale of 3–5. A score of 5 was for a constant exotropia, present 100% of the time during observation; a score of 4 was an exotropia present greater than 50% of the 30 seconds of observation, and a score of 3 was an exotropia present less than 50% of the 30 seconds of observation. If the participant did not have a manifest strabismus during the observation, the examiner then occluded one eye for 10 seconds and measured how long it took for the participant to recover fusion. The right eye was first occluded, then the left, and whichever took longer to regain fusion was repeated. The longest time taken to regain fusion of these three trials was used to determine the control score. A control score of 2 was given if recovery took more than five seconds, a control score of 1 was given if recovery took one to five seconds, and a score of 0 was given if recovery took less than one second. The OCS was performed at both distance and near three times throughout the examination: twice before the BEAM testing and once after. An average of three measurements was a standard way to evaluate IXT control due to the variability of the condition.^28^

The optometric exam including: Randot stereoacuity measured at near (40 cm) and distance (3 m), near point of convergence break, and negative fusional vergence and positive fusional vergence (PFV) measured using a 20/30 optotype at distance (6 m) and a 20/30 line of letters on a Gulden fixation target at near (40 cm). After completing the optometric measurements, the participants’ binocular eye position was objectively recorded during a 90-minute session using the BEAM while watching an age-appropriate movie selected by the participant, located 6 m away from the participant. After the BEAM recording, the OCS, UCT, and PACT were repeated at a distance and near.

## Results

### Participants

Twelve participants were recruited, all of similar age, with six having binocularly normal vision (five females; mean age with standard deviation [SD] = 1.7 ± 2.3 years) and six having basic type IXT (three females; mean age with SD = 9.8 ± 1.6 years). Each participant returned approximately two weeks later to repeat the BEAM recording and measurement of UCT, PACT, and OCS as described above. Both participants said the BEAM was comfortable to wear for the entire test session and was not too heavy on the nose or ears, similar to wearing spectacle lenses.

### Preliminary Eye Position Traces

**Figure 5.**
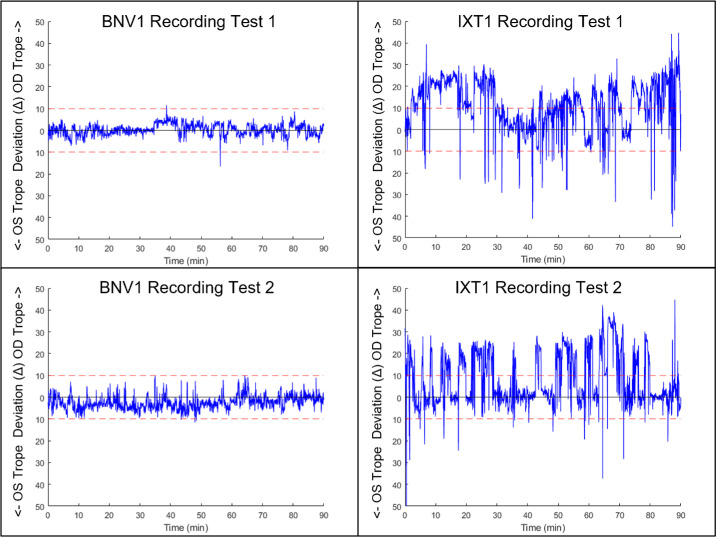
Comparison of a participant with BNV1 (*left*) and IXT1 (*right*) for two test sessions. Eye position (*blue solid line*) in prism diopters (pd) as a function of time (min). *Red dashed lines* are the clinical set threshold of ocular deviation, set here at 10Δ. Positive deviation is the right eye troping, and negative deviation is the left eye.

Figure 5 shows the eye movement recording from one typical participant with binocularly normal vision (BNV) and an individual with IXT for each of the two recording tests. The blue line represents the eye alignment in prism diopters (Δ) over time, with the threshold for deviation set at 10Δ (red dashed lines). The vertical axis shows the exodeviation of the right or left eye. Table 1 summarizes the data metrics for each participant. The office control score and Prism and Alternate Cover Test (PACT) are summarized in Table 2 below.

**Table 1.** Comparison of Ocular Deviation Between BNV Participants and IXT Participants With AVG and SD for Two Different Testing Sessions of 90 Minutes

Participant	Age	Testing Session	Deviation Time (Sec)	Deviation Time (%)	Maximum Deviation (Δ)	Mean Deviation (Δ)
BNV 1	14	1	0	0	0	0
		2	0	0	0	0
BNV 2	8	1	7	0	12	12
		2	0	0	0	0
BNV 3	10	1	5	0	12	12
		2	0	0	0	0
BNV 4	12	1	0	0	0	0
		2	0	0	0	0
BNV 5	13	1	29	1	20	14
		2	5	0	12	12
BNV 6	13	1	14	0	13	12
		2	1	0	13	13
BNV AVG ± SD	11.7 ± 2.3		5.1 ± 8.3	0.1 ± 0.3	6.8 ± 7.1	6.3 ± 6.3
IXT 1	8	1	2004	37	45	22
		2	2821	52	45	19
IXT 2	9	1	1079	16	42	16
		2	364	7	59	19
IXT 3	9	1	912	17	38	13
		2	657	12	28	13
IXT 4	12	1	1203	22	52	14
		2	1608	30	69	17
IXT 5	12	1	2131	39	69	17
		2	23	0	12	12
IXT 6	9	1	1009	19	50	17
		2	831	15	53	19
IXT AVG ± SD	9.8 ± 1.6		1220.2 ± 761	22.2 ± 14.2	46.8 ± 15.5	16.5 ± 2.9

AVG, averages.

**Table 2.** OCS and PACT Results for Each Participant

Participant	Testing Session	Office Control Score Near	Office Control Score Far	PACT Near (Δ)	PACT Far (Δ)
BNV 1	1	0	0	−1	−6
	2	0	0	−1	−6
BNV 2	1	0	0	0	−4
	2	0	0	0	0
BNV 3	1	0	0	0	−4
	2	0	0	0	−1
BNV 4	1	0	0	−3	−5
	2	0	0	−1	−4
BNV 5	1	0	0	0	0
	2	0	0	0	0
BNV 6	1	0	0	0	−2
	2	0	0	0	0
IXT 1	1	1.67	2.67	−32.5	−30.0
	2	1.67	2.67	−30.0	−30.0
IXT 2	1	4	4.33	−22.5	−35.0
	2	3.67	4.33	−27.5	−20.0
IXT 3	1	1	2.33	−27.5	−27.5
	2	1.67	1.33	−30.0	−17.5
IXT 4	1	2	3	−20.0	−22.5
	2	2.67	2	−20.0	−25.0
IXT 5	1	1.67	1.67	−45.0	−40.0
	2	1	1.67	−50.0	−40.0
IXT 6	1	2	1	−32.5	−30.0
	2	1	1.33	−30.0	−27.5

**Figure 6.**
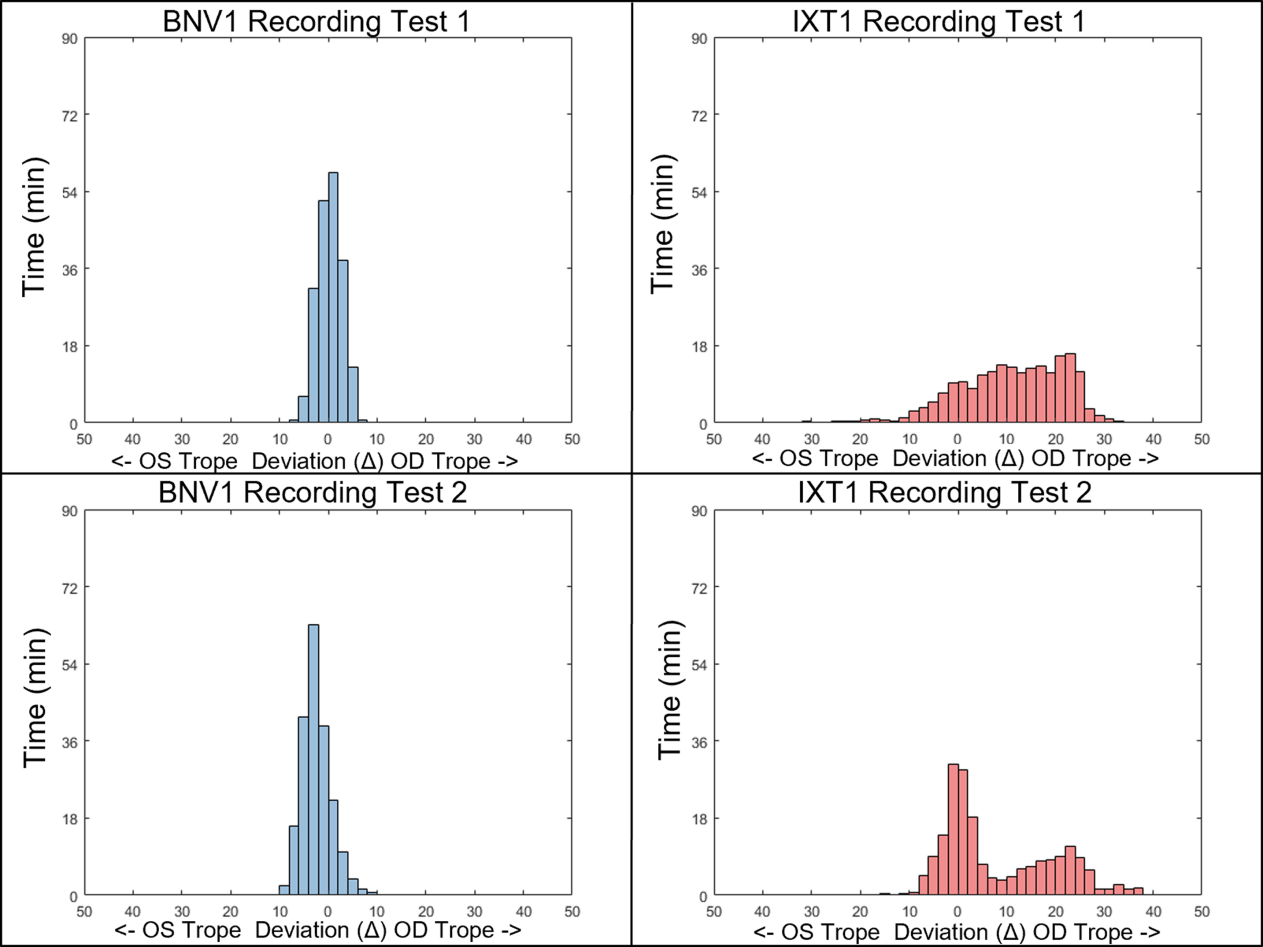
Histograms of the amount of time (min) of exodeviation for BNV (*left*) and IXT (*right*) participants for test 1 (*upper*) and test 2 (*lower*). The bin width is 2 prism diopter (pd) or Δ.

**Figure 7.**
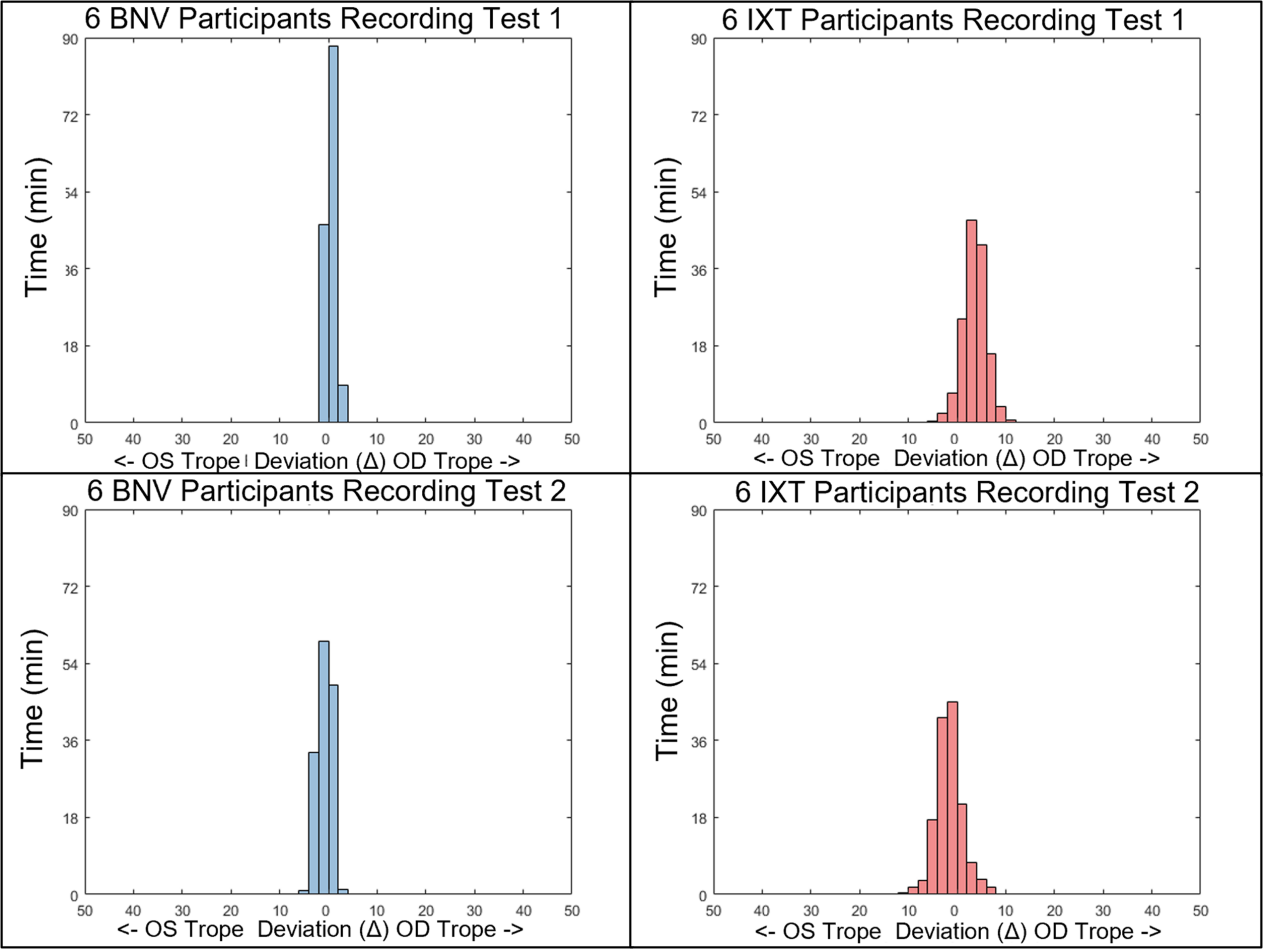
Histograms of average eye misalignment magnitudes for 6 BNV and 6 IXT participants, grouped at one-second intervals. *Bars* to the right of zero indicate right eye (OD) exodeviation, whereas *bars* to the left indicate left eye (OS) deviation. The bin width is 2 prism diopter (pd) or Δ.

A histogram of a single participant's deviations is shown in Figure 6. Two distinct patterns are observed between the participants (left plots compared to right plots). The IXT participant testing shows substantially more deviations outside of the threshold of 10Δ (blue trace deviating across the red dashed trace). Conversely, the BNV participant testing shows no deviations beyond the 10Δ. When comparing the first and the second day of testing, consistency is observed within the same participant (upper traces compared to lower traces). Figure 7 shows the histogram plots of six BNV (left column in blue) and six IXT (right column in red) participants for testing sessions 1 (top row) and 2 (bottom row).

## Discussion

The challenge of IXT is its intermittent nature; hence, the longer period of 90 minutes provides clinicians with objective data and analysis, compared to a clinical eye exam that examines eye rotation for only a few minutes. All 12 children, aged 8 to 14 years old, successfully completed the testing. The results support that the BEAM can objectively measure eye position and reveal distinct differences in ocular alignment patterns between individuals with BNV and those with IXT. All the results from these tests show a clear separation between the BNV and IXT groups. The BEAM offers the ability to objectively measure the following new metrics useful in diagnosing IXT: the time of deviation, the percentage of total test time deviated, and the maximum and mean deviation magnitudes. Future studies are planned to assess group-level population differences between BNV and IXT populations, with the IXT participating in therapeutic interventions, further investigating the capabilities of BEAM.

### Clinical Applications and Significance

Intermittent exotropia poses a unique diagnostic challenge to clinicians due to its variability. Clinicians typically observe a patient for only a few minutes during an examination. They may not be aware whether the observations made during the examination accurately represent the patient's control outside of the clinical setting. Studies have shown that the angle of the deviation can vary throughout the day when the exotropia is manifest.^29^^,^^30^ Beyond magnitude, variability has also been demonstrated in control of the IXT as measured with the Office Control Score, with variability occurring in as little as five minutes of observation.^28^ If a patient's presentation changes throughout the day, it is challenging for a clinician to assess the IXT or determine the effectiveness of a therapeutic intervention. Current diagnostic methods are limited to the clinic, significantly restricting the temporal range of testing. Hence, interpreting the effectiveness of surgical or nonsurgical interventions is challenging due to the limited time available for testing.

The comparison in Figure 5 between the IXT at each time point highlights the variability of an IXT patient's exodeviation when viewing the same visual task. In one instance, the IXT participant exhibits two or three periods of consistent exodeviation. During the next testing session, the participant deviates many more times, albeit for shorter durations at each exodeviation. When these results are compared to the clinical exam results, it highlights the challenge clinicians face. The control and deviation magnitude results are nearly identical, but the presentation differs, as shown by the objective measurements of the BEAM. Hence, it is unclear whether the difference in presentation is clinically meaningful between the two testing sessions based solely on clinical findings. Directly comparing the clinical examination result to the BEAM recording reveals slight differences in the OCS and magnitude of deviation. Converting the percentage of time deviated metric using the BEAM recording using the OCS guidelines, the first testing session has a grade of 4, and the second testing session has an OCS grade of 3. This difference highlights how the single time point measurements may not accurately capture the nature of intermittent exotropia. The converse occurs for the magnitude of deviation, with the mean deviation magnitude objectively measured being less than that measured by the PACT. The magnitude of deviation differences could be due to variability of the IXT magnitude or that the PACT measurements are dissociated, whereas the deviation objectively measured with the BEAM is spontaneous during viewing conditions, or associated conditions. This discrepancy could be due to differences in the testing method (associated versus dissociated) or may suggest that a non-dissociating method of deviation magnitude evaluation, such as the simultaneous prism cover test, may give a measured magnitude closer to that seen during the BEAM observation. Objective continuous monitoring methods have been discussed as important steps forward in IXT diagnostics.^5^^,^^31^ The BEAM provides a method to address this deficiency by quantifying ocular alignment without requiring a clinician to be present for an extended period.

Beyond diagnostics, the BEAM provides an unbiased, objective outcome metric. One issue in improving IXT treatment success rates is the inability to compare different randomized clinical studies, as many define success differently.^15^ Surgical intervention studies predominantly use the angle of deviation as their measure of success, which is challenging because surgery reduces the magnitude of deviation, making the size of the deviation smaller and more difficult to observe. During the 30-second observation period of the OCS, a deviation may manifest, but it may not be observed visually if it is small. Nonsurgical intervention studies predominantly use OCS as outcome measurements. However, nonsurgical methods do not aim to reduce the overall angle of deviation, but rather to improve control over exodeviation, thereby reducing its frequency of manifestation. Hence, surgical and nonsurgical interventions have different outcome measures, making it difficult to compare the effectiveness of each intervention. Both interventions also suffer from the variability of subjective outcome metrics, as these measurements must be taken by a human who might have unconscious or confirmation biases. The BEAM reduces bias, as a computer system is objective and cannot be influenced by bias or fatigue. Enabling more robust quantitative assessments of treatment options will allow researchers to compare the varied treatments for IXT using the same method of assessment, thereby improving treatment outcomes.

### Therapeutic Intervention

The BEAM platform has the potential to be integrated with current treatments, providing clinicians with more insight into how patients respond to different interventions. The BEAM's objective measures can provide assessment outcomes to determine effectiveness and modifications to therapeutic regimens. If included in a nonsurgical treatment protocol, the BEAM can provide real-time feedback to clinicians about how a patient responds to treatment, allowing for the potential application of personalized medicine to improve treatments.

Development has begun with a therapeutic version of the system. Prior research has shown that auditory biofeedback can improve oculomotor function and alignment, where an individual is presented with a sound to represent an unconscious oculomotor deviation.^32^ Auditory biofeedback has been tested in convergence insufficiency,^33^ nystagmus,^34^ and strabismus.^35^ All binocular dysfunctions have shown improvements after therapy. Auditory biofeedback systems were previously limited to expensive, bulky hardware, which restricted their feasibility for home use. Leveraging the BEAM platform to develop a therapeutic intervention would enable its use in many settings, including the home. Unlike the diagnostic methodology, the operator would set a deviation threshold before starting the therapy. Once the deviation exceeds this threshold, the system emits a tone, with its volume or frequency modulated by the size or temporal length of the deviation. Auditory biofeedback is hypothesized to reduce the suppression of the deviating eye, enabling the patient to manually realign the eye and improve fusional reserves, alongside recognizing visual suppressions. The BEAM could also augment existing vision therapy techniques, providing the patient with reinforcement when they complete different procedures within various therapeutic interventions.

### Study Limitations

Like all medical devices, the BEAM has its limitations. The BEAM may record a false-positive deviation, which can occur when a participant substantially turns their head while remaining visually fixated on the TV screen. Testing showed that when this happened, the ocular deviation typically did not exceed 10Δ. Hence, for this feasibility study, the threshold was set to 10Δ. During testing, there was only one observation of a deviation of more than 10Δ on the first day of testing, which lasted for a few seconds in the BNV participant within the 90-minute testing period. It was also observed that the BNV participant did not maintain 10Δ for extended periods, so a secondary threshold was set in the deviation detection algorithm to monitor 10-second ocular deviation. The longer deviation detection window reduces false-positive deviations caused by head rotation rather than ocular misalignment. Observing the variability in the BNV position trace, approximately 2Δ variability is observed, which is similar to the resolution of the current clinical methods.

## Conclusions and Clinical Translation

The BEAM represents a significant step forward in the diagnosis and objective assessment of IXT, providing a platform for the design of new therapeutic interventions. It will enable a better understanding of IXT under viewing conditions more like a home setting and offer the potential to compare treatment outcomes. The BEAM's novel set of objective metrics provides clinicians with further insight than the currently available tests, aiming to improve outcomes for IXT patients. This proof-of-concept device can be used as an objective outcome assessment for randomized clinical trials to assess and compare therapeutic interventions of IXT.
